# Decoding cellular plasticity and niche regulation of limbal stem cells during corneal wound healing

**DOI:** 10.1186/s13287-024-03816-y

**Published:** 2024-07-06

**Authors:** Di Sun, Xiaowen Zhang, Rong Chen, Tian Sang, Ya Li, Qun Wang, Lixin Xie, Qingjun Zhou, Shengqian Dou

**Affiliations:** 1 State Key Laboratory Cultivation Base, Shandong Provincial Key Laboratory of Ophthalmology, Eye Institute of Shandong First Medical University, Qingdao, China; 2 Qingdao Eye Hospital of Shandong First Medical University, Qingdao, China

**Keywords:** Cornea, Corneal epithelium, Limbal stem cells, Niche regulation, Single-cell RNA sequencing

## Abstract

**Background:**

Dysfunction or deficiency of corneal epithelium results in vision impairment or blindness in severe cases. The rapid and effective regeneration of corneal epithelial cells relies on the limbal stem cells (LSCs). However, the molecular and functional responses of LSCs and their niche cells to injury remain elusive.

**Methods:**

Single-cell RNA sequencing was performed on corneal tissues from normal mice and corneal epithelium defect models. Bioinformatics analysis was performed to confirm the distinct characteristics and cell fates of LSCs. Knockdown of *Creb5* and OSM treatment experiment were performed to determine their roles of in corneal epithelial wound healing.

**Results:**

Our data defined the molecular signatures of LSCs and reconstructed the pseudotime trajectory of corneal epithelial cells. Gene network analyses characterized transcriptional landmarks that potentially regulate LSC dynamics, and identified a transcription factor Creb5, that was expressed in LSCs and significantly upregulated after injury. Loss-of-function experiments revealed that silencing *Creb5* delayed the corneal epithelial healing and LSC mobilization. Through cell–cell communication analysis, we identified 609 candidate regeneration-associated ligand-receptor interaction pairs between LSCs and distinct niche cells, and discovered a unique subset of *Arg1*^+^ macrophages infiltrated after injury, which were present as the source of Oncostatin M (OSM), an IL-6 family cytokine, that were demonstrated to effectively accelerate the corneal epithelial wound healing.

**Conclusions:**

This research provides a valuable single-cell resource and reference for the discovery of mechanisms and potential clinical interventions aimed at ocular surface reconstruction.

**Supplementary Information:**

The online version contains supplementary material available at 10.1186/s13287-024-03816-y.

## Background

The cornea, as the transparent tissue on the ocular surface, is an important component of the visual system with significant refractive and barrier functions [1]. Wherein, the corneal epithelium located on the outermost layer of the cornea, is a stratified squamous non-keratinized layer, acting a key role in keeping the ocular surface moist and resisting infection, as well as preserving corneal transparency and optical function [2–4]. The corneal epithelium is highly regenerative and self-repairing, which depends on the proliferation and differentiation of limbal stem cells (LSCs) located in crypts along the cornea-scleral border [5]. And the integrity of the corneal epithelium is essential for a clear and stable vision.

Wound healing is a highly regulated process consisting of inflammation, epithelial reformation and regression stages [6]. The skin epidermis is a typical wound healing model, epidermal stem cells differentiate into terminally differentiated epidermal cells with important biological functions, thereby completing the process of epidermal wound healing [7, 8]. Analogously, when the corneal epithelium is damaged, LSCs change their cellular dynamics to differentiate into limbal progenitor cells and transit-amplifying cells in response to corneal epithelial cell renewal. These cells divide and migrate to the central basal layer of the cornea to promote corneal epithelial healing, thus reconstructing the ocular surface, restoring visual acuity and function of the eye [9–12]. This process is inseparable from the involvement of the LSC niche, also known as the surrounding microenvironment of LSCs. Previous studies revealed that various cell types, such as mesenchymal cells [13], immune cells [14], melanocytes [15], vascular cells [16] or even nerve fibers [17] and extracellular matrix and signal molecules [18], were involved in the functional regulation of LSCs as niche components. Once LSCs or their niche regulations performed abnormally, it may impede the wound healing process and pose a serious threat to vision.

Recently, various studies pertaining to the LSCs and their niches have revealed multiple new findings [19–21]. However, precisely how distinct transcriptional signals changed during wound healing, and which kind of niche cells participate in the regulation of LSCs, remain to be elucidated. High-throughput sequencing technology has allowed for the use of multiomics methods to study ocular tissues [22–25]. Recently, single-cell RNA sequencing (scRNA-seq) has emerged as a powerful tool that enables the investigation of previously unidentified cell types and the detailed analysis of their potential heterogeneity with unprecedented resolution [26–28]. Unlike traditional methods that analyze gene expression in the bulk-input tissues, scRNA-seq allows for the identification of subtle differences in individual cells [29–31], which help identify specific signals in different cell types and improve our understanding of the limbal stem cell dynamics and niche regulations. Single-cell based research and discoveries have been made in various eye tissues, including the cornea [32, 33], iris [34, 35], sclera [36, 37], uvea [38] and retina [22, 39, 40]. Recently, it was reported that mouse LSCs can be subdivided into quiescent LSCs (qLSC, located in the outer limbus) and active LSCs (aLSC, located in the inner limbus) [14]. Single-cell transcriptome can monitor specific transcription process in a cell type-specific manner [7, 41, 42]. Dissecting and studying the dynamic behaviors of LSCs and the molecular regulation mechanism of niches during corneal epithelial wound healing at the single-cell level was crucial.

Herein, we performed scRNA-seq analysis on mouse cornea from homeostasis and wound repair conditions, and compiled a transcriptomic atlas of all cell types originated from corneal tissues with an unprecedented resolution. Then we further divided corneal epithelial cells into 9 subpopulations and annotated through classical markers, including aLSCs and qLSCs, and determined the hierarchical characteristics and differentiation trajectories of these subpopulations during corneal wound healing. Then we mainly analyzed the specific changes in transcriptional regulons of LSCs and found that, *Creb5*, a core transcription factor in LSCs, play vital roles in the corneal epithelial regeneration, and knocking down *Creb5* delayed wound healing. In addition, we systematically depicted the cell–cell communications between LSCs and immune cells from limbal niche during corneal epithelial wound healing. Furthermore, we identified injury-induced *Arg1*^+^ macrophages could secrete OSM, thereby accelerating corneal epithelial wound healing. In conclusion, this study provided a comprehensive understanding of the LSC behaviors and their niche regulation mechanisms in homeostasis and regeneration at the single-cell level, and laid a foundation for further clinical interventions aimed at ocular surface reconstruction or other related disorders.

## Methods

### Mice

Male mice aged 6–8 weeks were procured from Vital River Laboratory Animal Technology Co., Ltd. (Beijing, China). They were housed at the animal center of the Shandong Eye Institute, adhering to specific pathogen-free (SPF) standard conditions including a temperature of 23 °C and 60% humidity. Throughout the experimental period, the mice had unrestricted access to both water and food. All animal experiments were in accordance with the Ethics Committee guidelines of Eye Institute of Shandong First Medical University (20201206-01). The approval number is SDSYKYJS No.20221009. All mice were euthanized by inducing anesthesia with 5% concentration of isoflurane, causing the mice to quickly lose consciousness and then subjected to cervical dislocation. All experimental procedures were approved by the Institutional Animal Care and Use Committee and adhered to the Association for Research in Vision and Ophthalmology (ARVO) Statement.

### Construction and injection of *Creb5*-targeting rAAV

AAV-*Creb5*-RNAi (GCAGTTGTTGTTAACACATAA) and AAV-NC at a titer of 1E + 12 v.g were applied topically by subconjunctival injection (5 μl per eye) to the 20 mice eyes to knockdown *Creb5* expression, after three weeks RNAs were extracted from the corneal epithelium of 5 mice to detect infection efficiency and the mouse corneal epithelial wound experiments were performed. All viruses were constructed by the Shanghai Genechem Co, LTD.

### Corneal wounding

Mice were subjected to anesthesia through intraperitoneal injection of pentobarbital sodium (50 mg/kg). Following this, procaine hydrochloride was applied topically to the ocular surface. The corneal central epithelium’s wound region in untreated normal mice was delineated using a trephine (2.5 mm diameter). Subsequently, the designated region was gently scraped using an Algerbrush II rust ring remover (Alger Co., Lago Vista, TX), with utmost care to avoid harm to the underlying corneal stromal layer. Sodium fluorescein was then employed to stain the wound area, facilitating the observation of healing kinetics. A slit-lamp microscope was used to capture images of the impaired zones. To ensure prevention of infection, the wound area was treated with ofloxacin. The same corneal epithelial debridement surgery was performed on 40 mice subconjunctiva injected with AAV-*Creb5*-RNAi and AAV-NC. Image J software (NIH, Bethesda, MD) was used to calculate the percentage of wound area (15 for each group).

### Treatment of recombinant-OSM

24 h before, 0 h after, and 24 h after performing mouse corneal epithelial wounding experiments, 100 ng/ml recombinant-OSM (Cloud-Clone Corp., Wuhan, China) were applied topically by subconjunctival injection (5 μl per eye) to the 20 mice eyes. The control group was saline. Image J software (NIH, Bethesda, MD) was used to calculate the percentage of wound area (20 for each group).

### Tissue dissociation and cell isolation

The eyes from 4 normal unwound (UW) mice and 4 mice 24 h post-wound (W) were collected to extract corneas. The corneas of mice underwent an 18-h digestion using dispase II (Roche) at 4 °C to facilitate the separation of the epithelial layer from other components. Subsequently, the epithelial layer underwent trypsin digestion (Sigma-Aldrich), while the residual tissue was subjected to collagenase A digestion (Roche) at 37 °C for a duration of 1 h [43]. Following this, the dissociated corneal cells from UW and W groups were individually pooled and re-suspended, in preparation for 10 × Genomics sequencing.

### 10× Genomics scRNA-seq

Single cells from every sample underwent separate processing to create single-cell suspensions and generate libraries using the 10× Genomics system. The cells were partitioned to generate GEMs, construct barcoded cDNA libraries, and were prepared using the single-cell 3′ mRNA kit (V2; 10× Genomics) as per the manufacturer’s instructions. Subsequently, all libraries underwent quality assessments (Fragment Analyzer 2100, Agilent Technologies), and sequencing was conducted (Platform: DNBSEQ; read length: 100 bp, paired-end).

### Data processing and downstream analysis

The transcripts were aligned with the appropriate reference genome (refdata-gex-mm10-2020-A for mouse) using the 10× Genomics CellRanger pipeline (version 3.1.0). Read count matrices were generated for each sample through CellRanger count. Subsequently, the count data were imported into the Seurat R package (version 3.2.2) [44]. To ensure library quality, the following steps were executed: Cells with gene counts falling below 500 or exceeding 8000, or exhibiting a mitochondrial gene ratio surpassing 10%, were excluded. Genes expressed in fewer than 5 cells were eliminated. Doublets were identified via the DoubletFinder package (version 2.0.3) [45]. The mean–variance-normalized bimodality coefficient (BCMVN) was computed for each sample to determine neighborhood size (pK_value), and the count of simulated doublets (pN_value) was set at 0.25. Accounting for dissociation-induced artifacts in sensitive cells, those expressing known dissociation-induced gene signatures were gradually identified and removed during analysis if no other explanatory marker genes were present [46]. Post the above filtering pipeline, the CCA method was applied to libraries from distinct experimental batches to mitigate batch effects in data integration [47]. Normalization employed the LogNormalize method, effectively addressing inherent variation stemming from mitochondrial gene expression. For cell clustering, a principal component analysis (PCA) focused on highly variable genes. Clustering, with a resolution of 0.4, was conducted for the top 15 principal components (PCs) using the graph-based shared nearest neighbor (SNN) technique (FindClusters function), yielding a total of 18 unsupervised cell clusters. Visualization of clustering results for individual or grouped samples utilized UMAP or t-SNE. Cell types were categorized through differential expression analysis, and cluster-specific marker genes were identified using the FindMarkers function. For the analysis of subtypes of CEpCs, the top 11 PCs were clustered using FindClusters and FindNeighbors functions to rearrange 16 unsupervised cell populations by UMAP. The top 13 PCs of ICs were clustered with a resolution of 1, and 13 unsupervised cell populations were re-clustered by t-SNE. The top 3 PCs of Monos were clustered with a resolution of 3, and 11 unsupervised cell populations were reclustered by t-SNE.

### Sample identity distribution

Sample identification and distribution of integrated corneal cells from 8 mice were performed using the Souporcell algorithm as previously described [48].

### RNA velocity analysis

The velocyto Python package was employed to recompute the counts of spliced and unspliced reads, utilizing the aligned BAM files. Subsequently, the SeuratWrappers and velocyto R package were utilized to compute RNA velocity values for each gene within every cell. These RNA velocity vectors were then integrated into the 2D diffusion map space [49].

### Identification of TFs using SCENIC

For the identification of active transcription factors (TFs) in aLSCs, qLSCs, MtCs, and CBCs, we conducted a single-cell analysis to infer TF networks using pySCENIC (version 0.10.3), following established protocols [50]. Comprehensive information on SCENIC can be accessed online at https://github.com/aertslab/SCENIC.

### Calculation of signature scores

Gene scoring analysis utilized gene sets sourced from the [8] genes list, with details provided in Additional file 1: Table S1. The calculation of signature scores for each gene set within every cell was carried out using the AddModuleScore function available in the Seurat R package. Subsequent significance testing was performed through a two-sided Wilcoxon rank sum test.

### Cell-cycle discrimination analysis

The determination of the cell cycle phase for each individual cell was executed within Seurat, leveraging cell-cycle-specific expression profiles [51]. In summary, G2/M and S phase markers were employed for scoring cells, while cells devoid of both G2/M and S phase markers were categorized as being in the G1 phase (CellCycleScoring function). Quantification of cells within each phase was achieved through utilization of the prop.table function.

### Pseudotemperal trajectory analysis

The SCORPIUS package (version 1.0.7) was employed to map cells onto pseudotime trajectories [52–55]. The analysis was conducted on genes characterized as highly variable, with the remaining parameters set to their default values. Following this, individual CEpCs within each subcluster were then positioned along linear pseudotime using the infer_trajectory function from the SCORPIUS package, utilizing the default settings. The R package Slingshot (version 1.4.0) was used to infer the differentiation trajectories of Monos and Macs, specifically by selecting *Ccr2*^+^ Macs as root cells after performing diffusion map dimension reduction and extracting pseudo-time values along the generated trajectories [56].

### Cell–cell communication analysis

We employed CellPhoneDB (version 1.1.0, https://github.com/Teichlab/cellphonedb) for systematic prediction of cell–cell interactions, utilizing ligand-receptor analysis with default parameters [57]. Subsequently, we focused on receptors expressed within ICs and ligands expressed in aLSCs/qLSCs, filtering those with a P-value < 0.05. Selected receptor-ligand pairs demonstrating significance were visualized using the Circlize R package to depict interaction links. To further predict active ligand-target associations contributing to LSCs, we utilized Differential NicheNet (https://github.com/saeyslab/nichenetr), an extension of the default NicheNet algorithm [58]. In this framework, aLSCs/qLSCs were designated as the “receiver/target” cell population within each niche, while ICs were labeled as the “sender/niche” cell population. By evaluating differential expression between niches and ligand activities, we prioritized ligand-receptor and ligand-target links. Among the top 20 ligands within the LSC niche, targets corresponding to these ligands with a score exceeding 0.25 were retained.

### Immunofluorescence staining and antibodies

Mice corneal tissues were rapidly frozen using Tissue-Tek Optimum Cutting Temperature Compound (Sakura Finetek, Tokyo, Japan). The frozen corneal Sects. (7 µm) were then fixed in 4% paraformaldehyde for a duration of 20 min. Following fixation, the sections were subjected to permeabilization, first with 0.1% Triton X-100 for 30 s, and subsequently with 1% Triton X-100 for 30 min. To prevent nonspecific binding, the sections were blocked with 5% BSA at room temperature for 1 h. For the immunofluorescence staining, primary antibodies were applied to the samples and left to incubate overnight at 4 °C. After thorough washing, the samples were exposed to fluorescein-conjugated secondary antibodies at 37 °C for 1 h. Subsequently, the stained sections were visualized using a positive inverted microscope (ECHO, LSM880) following counterstaining with 4′,6-diamidino-2-phenylindole (DAPI). The antibodies employed for immunofluorescence staining were anti-CREB5 (1:100, Thermo Fisher PA5-65,593), anti-Ki67 (1:200, Abcam ab16667), anti-GPHA2 (1:100, Santa Cruz sc-390194) and anti-OSMR (1:100, R&D MAB662). To label the secondary antibodies, Alexa Fluor 594-conjugated (1:400, Abcam ab150116) and 488-conjugated (1:400, Abcam ab150113) secondary antibodies were utilized.

### RT-qPCR

Total RNA was isolated utilizing the TransZol Up Plus RNA Kit (Transgen, Beijing, China). Subsequently, 1 mg of RNA was employed as a template for reverse transcription, employing random hexamer primers and the HiScript III RT SuperMix for qPCR (+ gDNA wiper) (Vazyme, Nanjing, China). The RT-qPCR process was executed utilizing the SYBR qPCR Master Mix (Vazyme, Nanjing, China) on a Rotor-Gene Q system (Applied Biosystems, Carlsbad, CA, USA). Each iteration of the experiment was independently replicated three times (n = 5). The analysis of relative gene expression data was conducted through the employment of the comparative CT method (ΔΔCT). The primer pairs (Deluohaida, Qingdao, China) employed for RT-qPCR were *Creb5* (5′–3′: TTCTGCCGTCTTGATGCCTAT 3′–5′: GTCAGCGCAGCCTTCAGTCT).

### Statistical analysis

Statistical analyses were performed using GraphPad Prism with t-test as appropriate. For gene set score analysis, statistical analysis was performed using two-sided Wilcoxon rank-sum tests. *P* values lower than 0.05 are considered statistically significant. *, **, *** and **** indicate *P* < 0.05, *P* < 0.01, *P* < 0.001 and *P* < 0.0001, respectively.

## Results

### Single-cell atlas of mouse cornea during wound healing

**Fig. 1:**
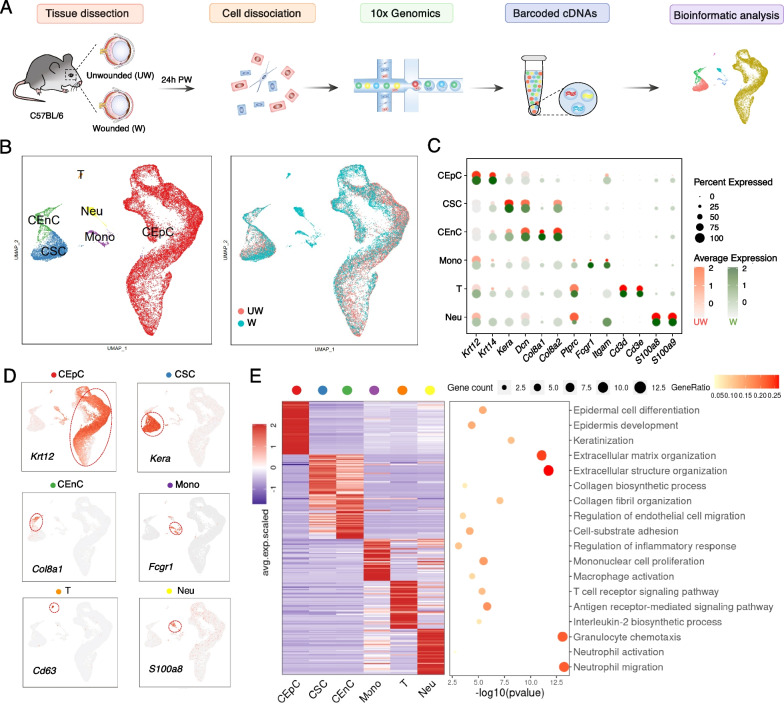
Cell types in mouse cornea identified by scRNA-seq analysis. **A** Flowchart overview of the scRNA-seq of unwounded and wounded mouse cornea. **B** UMAP plots showing cells colored by cell types (left) and groups (right). Abbreviations: CEpC, corneal epithelial cell; CSC, corneal stromal cell; CEnC, corneal endothelial cell; Mono, monocyte lineage; T, T cell; Neu, neutrophil; UW, un-wounded; W, wounded. **C** Dot plot showing high expression of classical marker genes for each cell type in two groups. Dots in red means UW and green means W. The color key from light to dark indicates low to high gene expression levels, and the dot size positively correlates with the percentage of cells positive for a given marker in a given type of cells. **D** Feature plots showing expression of classical genes for each cell types. The color key from light to dark red indicates gene expression levels. **E** Expression (left) and enriched GO terms (right) for top DEGs in each cell type. Each row represents one gene expression and each column represents one cell type, the value of each gene is row-scaled Z score

For a more detailed and comprehensive understanding of the process of mouse cornea during wound healing, we collected samples from 8 unwounded (abbreviated as “UW” in the following) and 8 wounded (abbreviated as “W”) mouse corneas and dissociated them to perform scRNA-seq using 10× Genomics platforms (Fig. 1A and Additional file 2: Fig. S1). After doublets removing and quality control, 21,400 cells from UW corneas and 15,967 cells from W corneas were generated, and an average of 2,882 genes and 22,751 transcripts were detected in each cell (Additional file 2: Fig. S2A). Then Seurat package [44] were used for unsupervised clustering and 17 clusters were identified (Additional file 2: Fig. S2B), with comparable contributions from all samples (Additional file 2: Fig. S2C) [48]. According to curated classical markers, we roughly distinguished six cell types, including corneal epithelial cells (CEpCs, *Krt12*^+^, *Krt14*^+^), corneal stromal cells (CSCs, *Kera*^+^), corneal endothelium cells (CEnCs, *Col8a1*^+^, *Col8a2*^+^), monocyte lineage (Mono, *Itgam*^+^, *Fcgr1*^+^), T cells (T, *Cd3d*^+^, *Cd3e*^+^) and neutrophils (Neu, *S100a8*^+^, *S100a9*) (Fig. 1B–D).

Furthermore, upon the analysis of gene ontology (GO) of differentially expressed genes (DEGs) for each cell types (Additional file 3: Table S2), the GO terms including “epidermal cell differentiation”, “extracellular matrix organization”, “regulation of endothelial cell migration”, “regulation of inflammatory response” all revealed the unique transcriptional features of each cell type separately, suggesting their unique biological functions (Fig. 1E). When we compared the cell constitutions between UW and W corneas, the proportions of CEpCs were decreased and immune cells were increased (Additional file 2: Fig. S2D). Overall, these results revealed corneal cellular heterogeneity in homeostasis and wound healing states and built a foundation for further research on the process of cornea wound healing.

### Hierarchy and differentiation trajectories of corneal epithelial cell subpopulations

**Fig.2:**
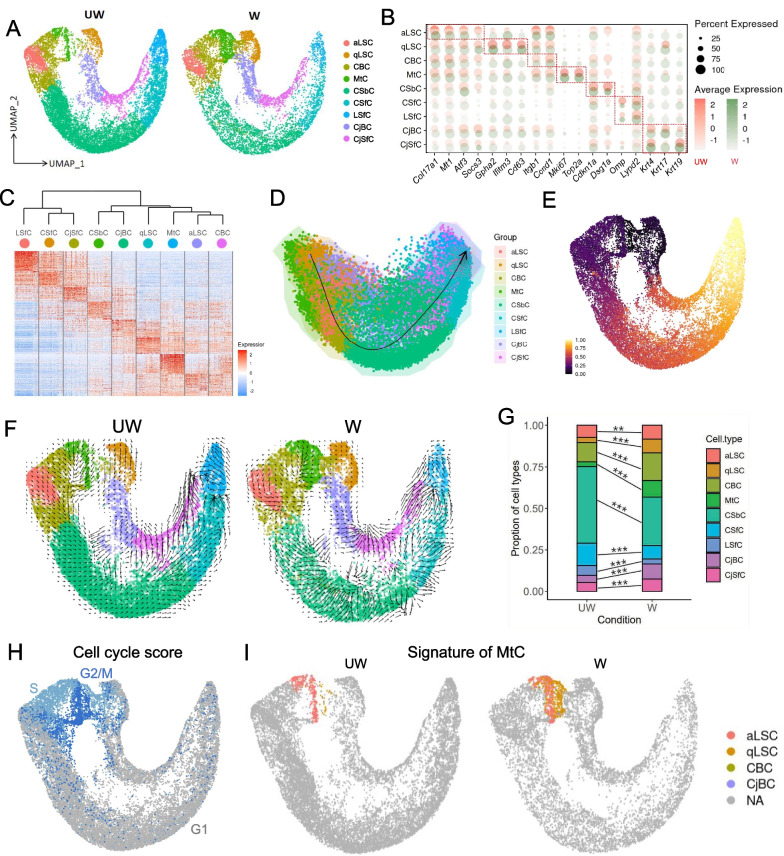
The heterogeneity and behaviors of corneal epithelial subtypes. **A** UMAP plots showing nine subtypes of corneal epithelial cells from UW and W groups. aLSC, active limbal stem cell; qLSC, quiescent limbal stem cell; CBC, corneal basal cell; MtC, mitotic cell; CSbC, corneal suprabasal cell; CSfC, corneal superficial cell; LSfC, limbal superficial cell; CjBC, conjunctival basal cell; CjSfC, conjunctival superficial cell. **B** Dot plot showing high expression of classical marker genes for each subtype in UW and W groups. **C** Heatmap showing the top DEGs of each corneal epithelial subtype. **D** Differentiation pseudotime trajectory of corneal epithelial subtypes calculated using SCORPIUS. **E** Projection of corneal epithelial cells pseudotemporal ordering analysis onto the UMAP space in Fig.S3A. Pseudotime order from black purple to bright yellow. **F** RNA velocity maps projecting onto the UMAP space in **A**. **G** Barplot showing the changes of corneal epithelial subtypes proportion between UW and W groups. The difference between the two groups was determined by chi-square test. **p < 0.01. ***p < 0.001. **H** Cell cycle score analysis revealed the signature of cells captured in specific stages of mitosis. **I** Cell type classification for MtCs shows that they consist of a mixture of cells with a hallmark of aLSCs, qLSCs and basal cells

LSCs with the capability of self-renewal and tissue regeneration play vital roles in wound healing process of the corneal epithelium [5]. To comprehensively understand the changes in cellular constitution and stem cell behaviors in response to corneal injuries, we first performed unsupervised sub-clustering on all corneal epithelial cells. Upon uniform manifold approximation and projection (UMAP) analysis [59] and cluster annotations using specific markers, corneal epithelial cells were divided into nine subpopulations in UW and W samples (Fig. 2A and Additional file 2: Fig. S3), including active limbal stem cells (aLSCs, *Col17a1*^+^ and *Atf3*^+^), quiescent limbal stem cells (qLSCs, *Gpha2*^+^ and *Ifitm3*^+^), corneal basal cells (CBCs, *Itgb1*^+^ and *Ccnd1*^+^), mitosis cells (MtCs, *Mki67*^+^ and *Top2a*^+^), corneal suprabasal cells (CSbCs, *Cdkn1a*^+^ and *Dsg1a*^+^), corneal superficial cells and limbal superficial cells (CSfCs, LSfCs, *Omp*^+^ and *Lypd2*^+^), conjunctival basal cells and conjunctival superficial cells (CjBCs, CjSfCs, *Krt4*^+^*, Krt17*^+^ and *Krt19*^+^) (Fig. 2B) [14]. Next, according to the differentially expressed genes for each subpopulation, we observed hierarchical similarities among several subpopulations, for instance, qLSCs, MtCs, aLSCs and CBCs were enriched in close branches, while cells in different state of differentiation were hierarchically distinct, such as superficial cells and suprabasal cells (Fig. 2C), consistent with the anatomic characteristics of corneal epithelium anatomy.

To further dissect the relationship of LSCs (aLSCs and qLSCs) and other corneal epithelial cells, pseudotime analysis using SCORPIUS [52–55] reconstructed the differentiation trajectory of corneal epithelium from LSCs to suprabasal cells and the terminally differentiated superficial cells (Fig. 2D, E), consistent with the principles of LSC differentiation. Next, for corneal epithelial cells, we performed RNA velocity analysis [60], which can estimate cell states by analyzing spliced and unspliced variants of mRNA in each cell. To decipher the plots of RNA dynamics, the state transition directions were indicated by arrows, and the extent of change were indicated by the arrows’ lengths. Compared with UW, we observed that aLSCs displayed larger RNA velocities in W group (longer and more arrows), demonstrating a rapid activation in cell state to initiate stem cell proliferation and differentiation, and similar or even stronger trend were observed in suprabasal and superficial cells, implying rapid cell migrations to response to wound recovery. However, it was noteworthy mentioned that qLSCs exhibited small velocities in W group (short or no arrows), suggesting a relatively inert response in 24 h post-wounding compared with aLSCs (Fig. 2F). We speculated that this situation may be related to the discrete dynamics of the two different LSC populations—the aLSCs located on the inner limbus differentiate into superficial cells to replenish the corneal epithelium more rapidly while the qLSCs located on the outer limbus expand into the central cornea during the wound healing gradually [14, 61]. Then when we surveyed the changes of cellular constitutions after corneal injury, we noted a significant increase of the proportions of aLSCs, qLSCs, CBCs and MtCs (Fig. 2G), which confirmed their essential roles in corneal wound healing process.

In addition, to clarify the main cell groups involved in corneal wound healing, we performed cell cycle analysis and found that MtC exhibited a signature of cells in S and G2/ M (Fig. 2H) and consisted of a mixture of cells with a hallmark (score of top ten genes) of all cell types (Fig. 2I). Surprisingly, the MtCs of the UW was predominantly derived from aLSCs and was located mainly in the S phase, while upon wound healing, a large number of qLSCs infiltrated and differentiated into MtCs of the G2/M phase, indicating qLSCs and aLSCs contributed differently during corneal epithelial wound healing.

Taken together, we identified various subpopulations involved in homeostasis and wound healing states of the corneal epithelium, and the differentiation trajectories among them were deciphered. We found that the behaviors of qLSCs and aLSCs in the process of corneal epithelial wound healing were different, laying a foundation to explore the alterations of LSCs in transcriptional profiles during wound healing of corneal epithelium.

### Subpopulation-specific changes of corneal epithelium during wound healing

**Fig.3:**
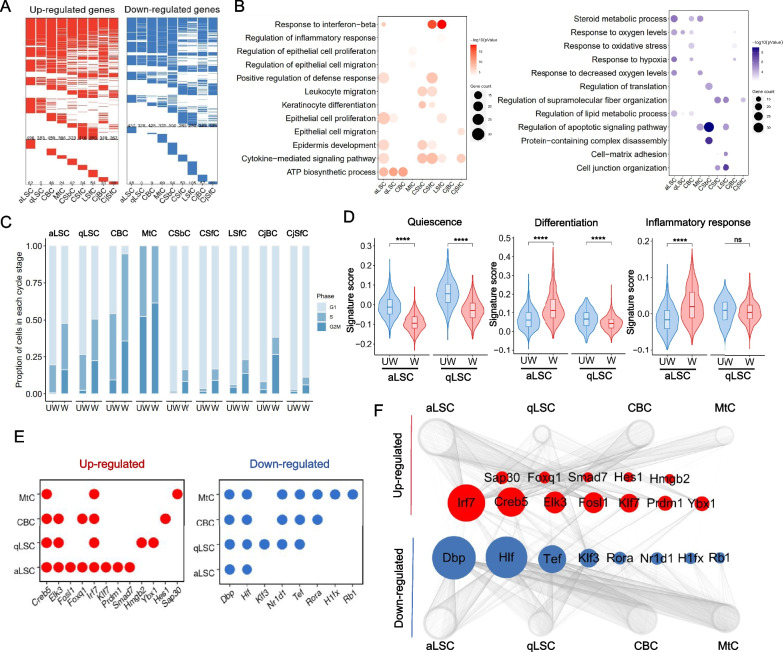
Alterations and differences in transcriptional profiles of nine subpopulations of corneal epithelial cells during wound healing.** A** Heatmaps showing the up-regulated (red, left) and down-regulated (blue, right) DEGs in nine subtypes of corneal epithelial cells between W and UW groups. Color white represents genes without differential expression. The number of DEGs are indicated on the maps. The part above the dotted line indicates DEGs are shared by at least two cell subtypes, and the part below the dotted line indicates DEGs are unique to each cell subtype. **B** Dot plots showing the representative GO terms of up-regulated (red) and down-regulated (blue) DEGs in each corneal epithelial cell type. **C** Barplot showing the proportion of each subpopulation of corneal epithelial cells at the cell cycle stage in both UW and W groups. **D** Gene signature scoring analysis across aLSC and qLSC in both UW and W groups using quiescence, differentiation and inflammatory response related genes. ****P < 0.0001 (two-sided Wilcoxon rank-sum test). **E** Dot plots showing up-regulated (red) and down-regulated (blue) core regulatory TFs of aLSC, qLSC, CBC and MtC. **F** Visualized network showing up-regulated (red) and down-regulated (blue) core regulatory TFs of aLSC, qLSC, CBC and MtC. The size of nodes is positively correlated with the number of edges

Next, we surveyed the alterations in transcriptional signals in each subpopulation of corneal epithelium during wound healing. We compared the gene expression programs for each subcluster between W and UW corneal epithelium, and identified 3,746 up-regulated genes and 3,446 down-regulated genes that were differentially expressed in at least one subcluster of corneal epithelium. Among these DEGs, approximately 90% of them were shared by at least two epithelial subtypes, implying similar responses across various subpopulations to corneal injury (Fig. 3A and Additional file 4: Table S3). Upon GO analysis, the core biological processes annotated for up-regulated DEGs were involved in epithelial cell differentiation and proliferation, cell cycle and inflammatory response, while down-regulated DEGs were enriched for catabolic and apoptotic process (Fig. 3B).

We then profiled the cell cycle state of each corneal epithelial subclusters in UW and W groups. During wound healing, almost all epithelial cells, especially for aLSCs, qLSCs, CBCs cells in G1 phase were obviously decreased, while cells in S and G2/M phases were increasing; for MtCs, cells in S phase were prominently decreased and more cells entered G2 phase (Fig. 3C), suggesting diminishing quiescence and activated cell division of these cells after injury, consistent with our previous observations (Fig. 2F). Then we focused on the status change of limbal stem cells, including aLSCs and qLSCs, and performed gene set score analysis for quiescence, differentiation and inflammatory response (Additional file 1: Table S1). We noted that when injury occurred, aLSCs and qLSCs uniformly scored lower for quiescence, while significant higher differentiation and inflammatory scores were observed in aLSCs (Fig. 3D). Altogether, these data demonstrated that corneal epithelial cells dramatically upregulate migration- and inflammation-associated genes’ expression during wound healing, especially in aLSCs, which existed in a lower-quiescence and higher-differentiation states compared with qLSCs, consistent with the cell traceability of MtCs (Fig. 2H, I).

Furthermore, to predict the core transcription factors (TFs) related to corneal wound healing, we performed the single-cell regulatory network inference and clustering (SCENIC) [50] analysis for aLSCs, qLSCs, CBCs and MtCs (Additional file 2: Fig. S4), and screened the top 10 TFs expressed in each cell type (Fig. 3E, F). We found several TFs, such as *Irf7, Creb5, Elk3, Fosl1* and *Klf7*, appeared in the top up-regulated genes in four types cells. *IRF7* is a member of the interferon regulatory factors (IRFS) family and participates in type-I interference (IFN) signaling, and plays a role in antigen presentation function of human corneal endothelial cells [62, 63]. The latest research showed that *FOSL1* as the human LSC-specific TFs that determined the LSC fate and was important in epidermal cells [64]. Furthermore, *KLF7* promoted the corneal progenitor cell state [65]. In summary, these TFs suggested a correlation with corneal healing.

### *Creb5* as a core transcription factor of LSCs exerted promotion effects on wound healing

**Fig.4:**
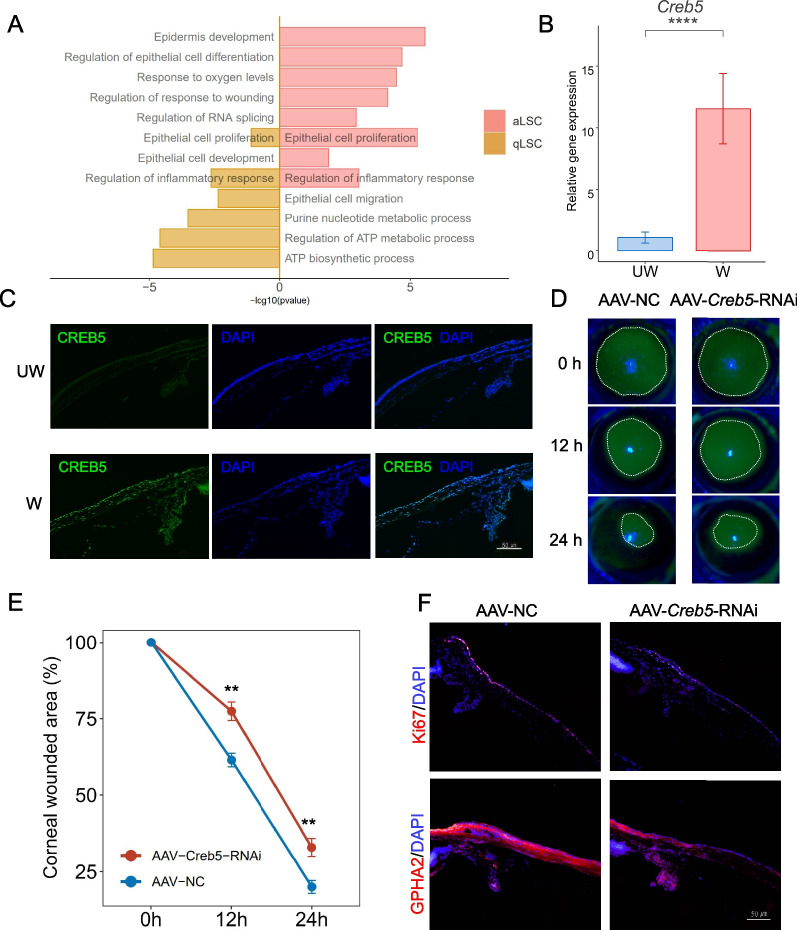
Knockdown of *Creb5* in mouse corneal epithelium.** A** Barplot showing the representative GO terms of *Creb5*-target genes of aLSC and qLSC.** B** Barplot showing the expression of *Creb5* in UW and W mice corneal epithelium quantified by RT-qPCR. ****P < 0.0001, t test.** C** Immunofluorescence staining showing the expression of CREB5 in UW and W mice healing corneal limbus. **D** Fluorescent dye staining showing the wound healing at 0, 12 and 24 h after corneal epithelial debridement in mice injected with AAV-NC and AAV-*Creb5*-RNAi*.* **E** Line chart showing the rate of epithelial healing in AAV-NC and AAV-*Creb5*-RNAi. **P < 0.01, t test. **F** Immunofluorescence staining showing the expression of Ki67, GPHA2 in mice injected with AAV-NC and AAV-*Creb5*-RNAi healing corneal limbus

Among these TFs, we noted that only *Irf7* and *Creb5* can be identified in all four subtypes, suggesting their core functional roles in corneal injury recovering. While we noted that solely *Creb5* were specifically expressed in limbal stem cells and basal epithelial cells (Additional file 2: Fig. S5A, B), suggestive of the potential involvement of *Creb5* in the promotion of corneal epithelial wound healing. Existing research showed that *Creb5* had crucial roles in regulating cell growth and proliferation [66–68], but there is no relevant report in corneal epithelial wound healing. We performed GO analysis on the target genes of *Creb5*, noticing that these GO terms enriched in epithelial cell proliferation and development (Fig. 4A). Surprisingly, *Creb5* was not only specially expressed in aLSCs, qLSCs, CBCs and MtCs of mice (Additional file 2: Fig. S5A), but its ortholog CREB5 were also detected in the human limbal stem/progenitor cells (LSPC) (Additional file 2: Fig. S5C–E) [16]. And we experimentally confirmed that *Creb5* expression increased after corneal epithelial wound in normal mice (Fig. 4B, C). To identify the potential role of *Creb5* for corneal epithelial wound healing, we conducted AAV mediated *Creb5*-knockdown (AAV-Creb5-RNAi, U6-MCS-CAG-EGFP) experiments in mice to observe the rate of corneal damage repair (Additional file 2: Fig. S5F). We performed corneal epithelial debridement in mice, and fluorescence staining showed delayed repair of corneal epithelium in mice injected subconjunctival with AAV-*Creb5-*RNAi compared with injected subconjunctival NC sequence-loaded virus (AAV-NC, U6-MCS-CAG-EGFP) (Fig. 4D, E), indicating *Creb5* played an important role in corneal epithelial healing. Furthermore, immunofluorescence staining analysis validated the effectiveness of AAV-*Creb5-*RNAi sequence loaded virus which interfered with Gpha2/Ki67 signal expression of LSCs compared with AAV-NC (Fig. 4F and Additional file 2: Fig. S5G), suggesting that knocking down *Creb5* leads to a decrease in the stemness and proliferative capacity of LSCs. In conclusion, *Creb5*, as a TF of LSCs, regulating LSCs to promote epithelial repair during corneal epithelial wound healing. These results provide a new insight for the discovery of new therapeutic targets and clinical drugs to promote corneal epithelial repair.

### Infiltrated immune cells during wound healing

**Fig.5:**
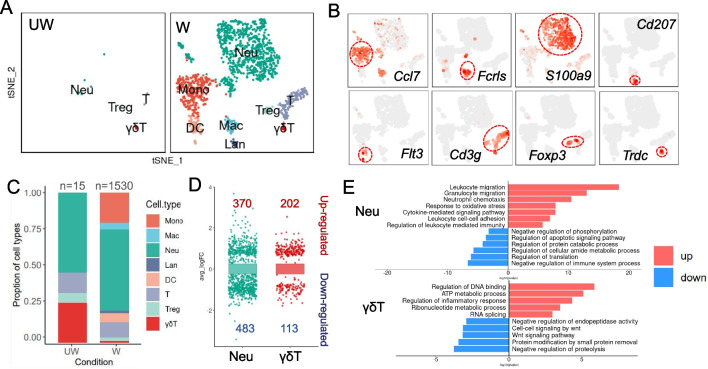
Immune cells in mice cornea during wound healing. **A** The t-distributed stochastic neighbor embedding (t-SNE) plots showing immune cells from UW and W groups. Mono, mononuclear cell; Mac, macrophage; Neu, neutrophil; Lan, Langerhans cell; DC, dendritic cell; Treg, regulatory T cell; γδT, γδT cell. **B** Feature plots showing expression of classical marker genes for immune cells. The color red indicates high gene expression levels. **C** Barplot showing the proportional changes of immune cells between UW and W groups. **D** Differential gene expression analysis showing up- and down-regulated genes in Neu and γδT in W group. **E** Barplots showing the representative GO terms of up-regulated (red) and down-regulated (blue) DEGs in Neu and γδT

The cornea is an immune privilege, with only a small number of immune cells in corneal homeostasis, but we found a large number of immune cells infiltration into cornea during wound healing (Additional file 2: Fig. S2D), so we performed unsupervised sub-clustering analysis on all immune cells to further explore their composition and transcriptional signals. Upon t-distributed stochastic neighbor embedding (t-SNE) analysis [44] and the known cell type markers’ annotation (Additional file 2: Fig. S6), we identified 4 and 8 cell clusters in UW and W samples (Fig. 5A), respectively. Feature plots of key cell type markers revealed population-level changes in mononuclear cells (Monos, *Ccl7*^+^), macrophages (Macs, *Fcrls*^+^), neutrophils (Neus, *S100a9*^+^), langerhans cells (Lans, *Cd207*^+^), dendritic cell (DCs, *Flt3*^+^), T cells (*Cd3g*^+^), regulatory T cells (Tregs, *Foxp3*^+^) and γδT cells (γδT, *Trdc*^+^) (Fig. 5B). We noticed the immune cell number of UW sample was only 15, while the W sample had 1,530 immune cells (Fig. 5C). The numbers of various cell types were increased, among them, Monos, Macs, Lans and DCs only appeared during wound healing, implying their crucial roles in promoting damage repair.

In addition to changes in cell composition, we further want to explore changes in gene expression in immune cells. However, owing to the too few cells of some particular cell types detected in UW sample, we only compared the gene expression programs for Neus and γδT between UW and W samples, and identified 853 and 315 DEGs in Neus and γδT separately (Fig. 5D), suggesting their potential regulatory roles in corneal wound healing process, though detailed mechanisms need to be further explored. Furthermore, upon GO analysis, the core biological processes annotated for up-regulated DEGs were involved in inflammatory response, immune cells migration and energy metabolic process (Fig. 5E), which means the process of corneal epithelium wound healing was regulated by immune cells.

### Cell–cell communications between LSCs and immune cells during corneal wound healing

**Fig.6:**
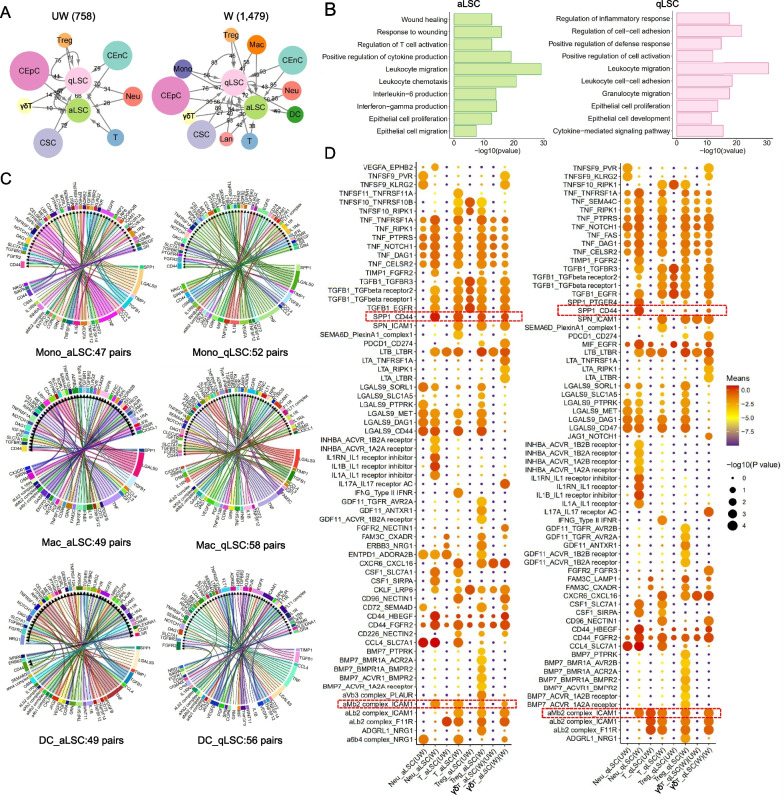
Changes in cell–cell communications between LSCs and immune cells during wound healing. **A** Visualized networks showing the number of regulatory effects of other corneal cells on LSCs in UW (left) and W (right) groups. Node size represents the number of ligand-receptor pairs. **B** Barplots showing the representative GO terms of target genes of increased Immune-LSC pairs. **C** Chord plots showing cellular interactions between Mono/Macs/DCs and aLSCs (up)/qLSCs (down), separately. The cell types and interaction pairs number are labeled. **D** Dot plots showing the ligand-receptor interactions associated with aLSCs (left)/qLSCs (right) in W group compared to that in UW group. aLSCs/qLSCs express receptors and receive ligand signals from Neus, T, Tregs and γδTs. Rows represent ligand-receptor pairs, and columns represent interactions between cells. The samples of UW or W are labeled in parentheses. The P value and means are calculated by the CellphoneDB analysis. Deep color represents high means, large circle represents high P value

Current research evidenced that T cells as the niche cell of qLSCs had a vital effect on maintaining quiescence and controlling the thickness of epithelial in homeostasis [14]. However, the potential roles of other cells in the limbal niche in regulating LSCs during wound healing were still elusive. To further explore the niche regulation relationship between LSCs and other cells during wound healing, we performed cell–cell communications analysis of UW and W groups using CellphoneDB. As shown in networks (Fig. 6A and Additional file 2: Table S4), from UW to W, the number of the interaction pairs identified between LSCs (aLSC and qLSC) and other niche cells was increased from 758 to 1,479, signifying that the cell–cell communications were enhanced, and more cells participated in the niche regulation of LSCs during wound healing. Since the increased proportion of immune cell interactions number is the highest, we next focused on the regulation of immune cells on LSC. Therefore, we selected increased ligand-receptor pairs genes between ImCs and LSCs for GO analysis (Fig. 6B) and found that, terms were highly related to inflammation response, epithelial cell proliferation and development function. Besides, we also structured the cell–cell interaction maps of ImC-LSC pairs (Additional file 2: Fig. S7A) and found the pairs associated with four cell types (including DCs, Macs, Monos, Lans) increased in W group had the largest number, indicating their nonnegligible roles in regulating aLSC and qLSC functions.

In order to seek the detailed interactions between these four gained cell types and LSCs in W, we detected the ligand-receptor pairs between them (Fig. 6C and Additional file 2: S7B). We observed that, some classical signaling such as *TNF-NOTCH1* pair was expressed in all eight gained ImC-LSC, as reported, Notch and TNF signaling had all been verified to regulate the homeostasis of the corneal epithelium and the corneal inflammatory response and wound healing after injury [69–72]. Strikingly, we found that *LGALS9* is also fully expressed and regulates multiple receptors of LSCs. As an epithelial repair modulator, *LGALS9* is reported to be involved in the regulation of cell proliferation and epithelial recovery after intestinal epithelial injury [73], but its specific functions in corneal epithelium repairing need further investigations. Moreover, *CX3CR1* ligand in Mac and Lan can promote epithelial repair [74], *JAG2-NOTCH1* signaling in DC can regulate epithelial differentiation and proliferation [75]. All these data evidenced that immune cells play an important role in regulating stem cells and promoting epithelial repair.

Though Neu, T, Treg and γδT were all detected in the UW and W, we still explored their changes in intercellular signaling, and identified significant alterations in the ligand-receptor pairs (Fig. 6D). For instance, *aMb2 complex-ICAM1* signaling in all cell pairs remarkably increased, representing that the signaling axis greatly participated in and intervened with the behaviors of LSCs. Studies had reported *aMb2* can regulate more precisely Neus activities [76] and *ICAM1* can promote wound healing by promoting γδT cells to migrate to corneal epithelial cells [77]. Besides, the signal pairs involved by *TGF-β* obviously enhanced during wound healing. TGF-β pathway, one of the most important and classical pathways for niche regulation of LSCs, can promote cell migration and inhibit cell proliferation, results in leading to a rapid initial regeneration of the corneal epithelium [78–80]. Notably, we observed that *SPP1-CD44* pair was more influential on aLSC than on qLSC, with its enhanced signal in all four immune cells. *SPP1* (Osteopontin), a secreted phosphoprotein 1, can regulate immune function, vascular remodeling, wound healing and developmental. *SPP1* combined with *CD44* can regulate signaling cascades that impacted processes including adhesion, migration, invasion, chemotaxis, and cell survival [81]. Nevertheless, the current research on *SPP1* was merely carried out in tumor, and no relevant studies can be found in the cornea direction. Consequently, we speculated that *SPP1-CD44* acted as an inducer to regulate aLSCs in order to actively respond to the signals of wound healing and participate in corneal epithelial reconstruction.

**Fig.7:**
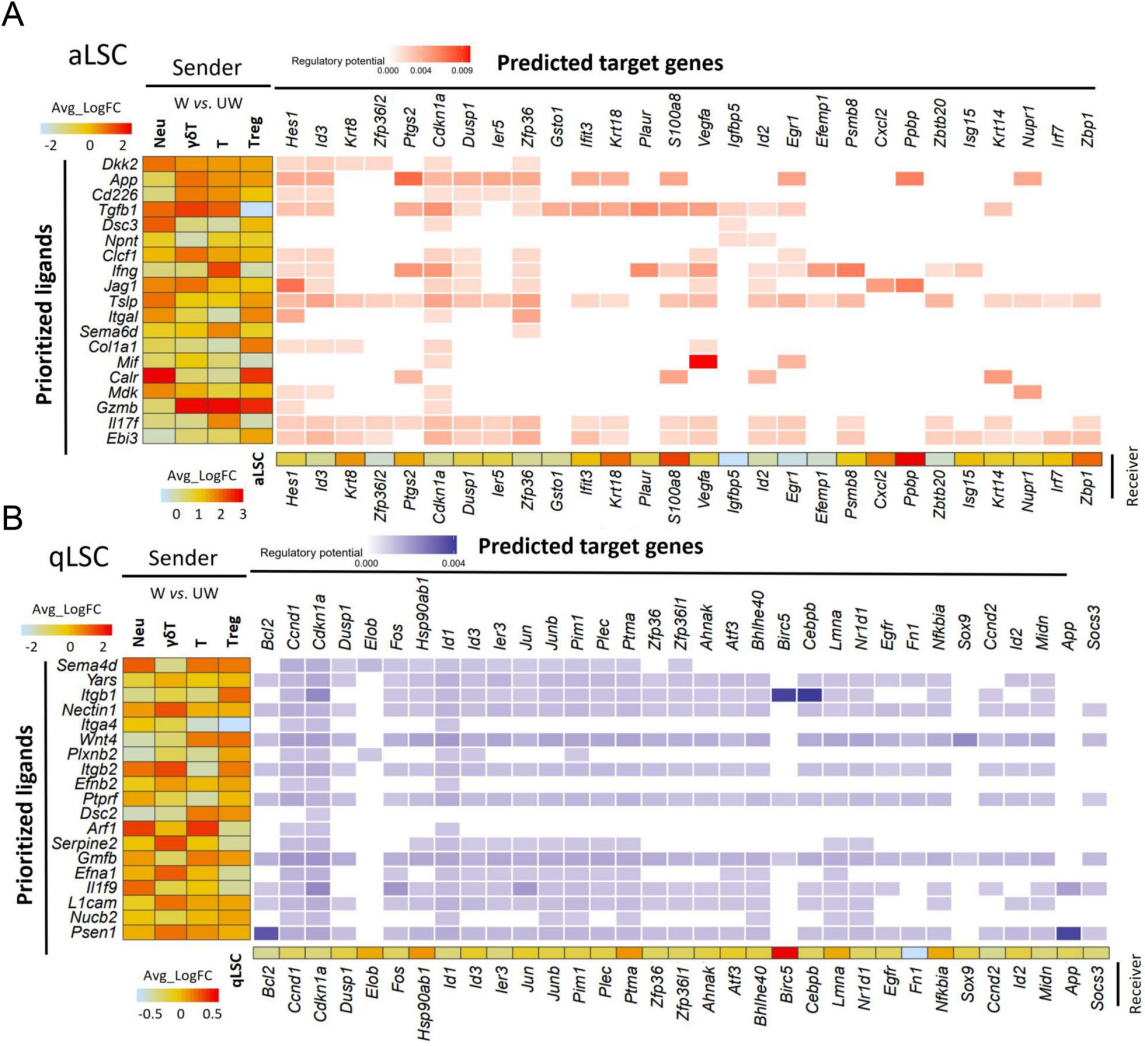
NicheNet analysis between increased immune cells and LSCs during cornea wound healing. **A** NicheNet analysis showing the interaction between Neus/T/Tregs/γδTs and aLSCs. Middle, heatmap predicting ligand-target regulatory potential. Left, heatmap predicting the average log_2_FC of the top ligands’ expression between UW and W groups for Neus/T/Tregs/γδTs. Bottom, heatmap predicting the average log_2_FC of ligand-matched targets expression between UW and W groups for aLSCs. **B** NicheNet analysis showing the interaction between Neus/T/Tregs/γδTs and qLSCs. Middle, heatmap predicting ligand-target regulatory potential. Left, heatmap predicting the average log_2_FC of the top ligands’ expression between UW and W groups for Neus/T/Tregs/γδTs. Bottom, heatmap predicting the average log_2_FC of ligand-matched targets expression between UW and W groups for qLSCs

Additionally, to further investigate the potential role of Neu, T, Treg and γδT cells in the process of regulating the involvement of LSCs in corneal epithelial wound healing, we performed NicheNet analysis on aLSC and qLSC respectively [58], allowing us to predict the interactions by linking ligands in Neu, T, Treg and γδT cells and the target genes in LSCs (Fig. 7A, B). Nichenet analysis predicted that γδT-derived *Jag1* may induce the expression of *Hes1* in aLSCs. *Jag1*, a characterized ligand for *Notch1* and *Notch2* receptors [82, 83], and *Hes1* also participated in NOCTH signaling as a target gene and can regulate corneal epithelial stem/progenitor cell homeostasis [84], suggesting *Jag1-Hes1* interaction can jointly regulate Notch pathway, activate the proliferation of aLSCs, and promote the repair of corneal epithelial. Interestingly, we observed that *Ptgs2* expressed in aLSC was associated with the γδT-derived *App*. *Ptgs2*, known as cyclooxygenase 2 (*Cox2*), is rapidly expressed in a variety of cell types in response to growth factors, cytokines, and proinflammatory molecules [85, 86] and can induce cancer stem cell-like activity and promote the proliferation, inflammation, invasion, and metastasis of cancer cells [87]. It has been reported that *TGFbeta-1* improved ovarian surface epithelium cells survival rate by activating *Cox2* to promote ovulation wound repair [88]. In addition, Treg cells as a niche cell of qLSCs [14], we detected that Itgb1, a ligand of Treg-derived, interacted with qLSC and activated the high expression of target genes *Birc5* and *Cebpb*. *Birc5* (Survivin) served the dual functions in preventing cell apoptosis and promoting proliferation [89, 90], and *Cebpb* also acted a crucial role in promoting the proliferation and differentiation of hematopoietic stem cells/progenitor cells [91] and breast stem cells [92], and regulating the stemness of enamel epithelial stem cells [93].

Overall, we systematically analyzed the cell–cell communications between all immune cells and LSCs during corneal epithelial wound healing for the first time, providing reference bases for the involvement of LSCs in reconstructing the ocular surface.

### OSM derived from injury-induced Arg1^+^ macrophages promote corneal epithelial repair

**Fig.8:**
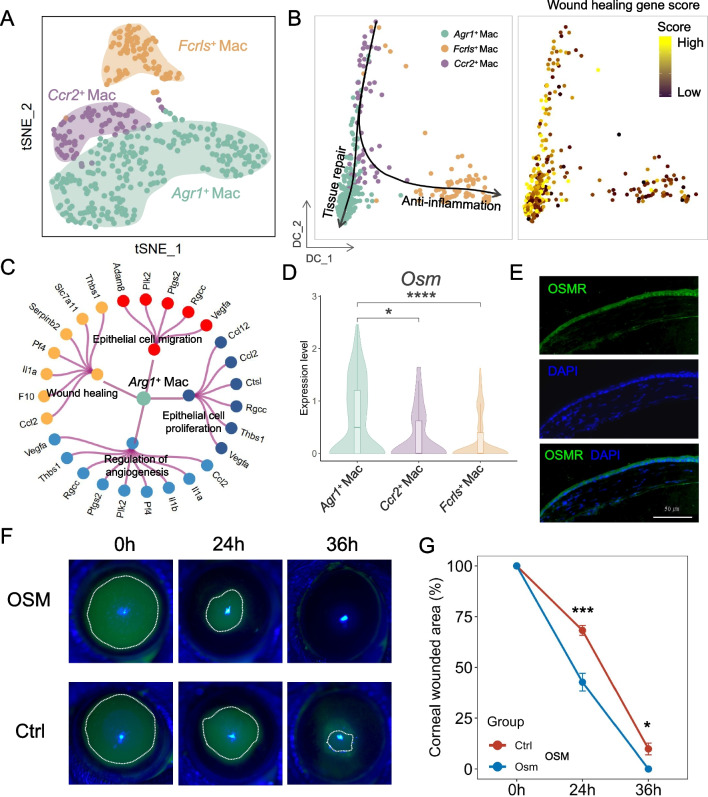
The burst of *Arg1*^+^ Macs during corneal wound healing**. A** t-SNE plot showing the distribution of three subpopulaitons of Monos and Macs. **B** Diffusion map showing the distribution of Macs subpopulations (left) and the scores of wound healing-related genes (right). **C** Network showing the enriched GO terms of *Arg1*^+^ Mac marker genes. **D** Violin plot showing the expression levels of *Osm* in Macs subpopulations. *P < 0.05, ****P < 0.0001, t test. **E** Immunofluorescence staining showing the expression of OSMR in corneal limbus. **F** Fluorescent dye staining showing the wound healing at 0, 24 and 36 h after corneal epithelial debridement in mice injected with Ctrl and Osm*.* **G** Line chart showing the rate of epithelial healing in Ctrl and Osm. *P < 0.05, ***P < 0.001, t test

Importantly, the appearance of Monos and Macs weas induced during corneal epithelial wound healing (Fig. 5C), indicating that Monos and Macs would play an important role in wound healing. Attracted by this finding, we performed t-SNE analysis and subdivided these cells into three subpopulations, including *Arg1*^+^ Macs, *Ccr2*^+^ Macs and *Fcrls*^+^ Macs (Fig. 8A and Additional file 2: S8A, B). Among them, not only the expression of *Ccr2*^+^ was highly expressed in Pre-Mono [94], but also *Ccr2*^+^ Macs had phenotypical similarities to M1-polarized macrophages [95, 96], suggesting that *Ccr2*^+^ Macs were both a population of precursor cells and had a pro-inflammatory effects. Consequently, to better understand the characteristic and differentiation trajectory of Macs, we analyzed their expression patterns. We revealed the distribution of three subpopulations using *Ccr2*^+^ Macs as root cells by diffusion map dimensionality reduction analysis and identified *Arg1*^+^ Macs was particularly characterized by wound healing (Fig. 8B). In addition, these *Arg1*^+^ Macs showed elevated expression of genes associated with wound healing, epithelial cell proliferation, regulation of angiogenesis and epithelial cell migration (Fig. 8C).

Intriguingly, we noticed *Osm*, as a ligand in the ligand-receptor pairs of ICs-LSCs (Fig. 6C), was highly expressed in *Arg1*^+^ Macs (Fig. 8D). Furthermore, we found that *Osmr* as its receptor was also expressed in the corneal epithelium, and its ortholog *OSMR* were also detected in the human corneal epithelium (Fig. 8E and Additional file 2: S8C, D). Previous studies have shown that OSM, as a secreted niche factor of ICs, played a momentous role in wound re-epithelialization and stem cell quiescence and stemness [97–100]. To investigate the effect of *Osm* on the corneal epithelial wound healing, we subconjunctivally injected recombinant-OSM for the treatment of corneal epithelial wound in mice, which was shown to accelerate wound healing (Figs. 8F, G). Taken together, wound healing-induced *Arg1*^+^ Macs could secrete OSM, thereby accelerating corneal epithelial wound healing.

## Discussion

Here, we reported a comprehensive single-cell compendium of the behaviors of aLSCs and qLSCs and their niche regulation during corneal epithelial wound healing in mice at the molecular level. We observed changes in gene expression, transcriptional regulation, and cell–cell communication of aLSCs and qLSCs between UW and W samples, which together provided insight into the mechanisms of the LSCs involvement in the repair of corneal epithelium. More precisely, knockdown of key TFs involved in corneal epithelial wound healing, such as *Creb5*, which is specifically expressed in LSCs, delayed corneal epithelial wound healing. Next, we mapped the atlas of the cell–cell communications between immune cells and LSCs and identified some ligand-receptor pairs associated with wound healing. In addition, we found wound healing-induced *Arg1*^+^ Macs could secrete *Osm*, thereby accelerating corneal epithelial wound healing. In conclusion, these findings provide new understanding of the involvement of LSCs in corneal epithelial wound healing and define new biological targets for the treatment of clinical diseases associated with corneal epithelial wound.

As outlined in preceding studies, limbal stem cells exhibit heterogeneity. The outer limbus hosts quiescent LSCs (qLSCs) while inner LSCs actively renew the cornea (aLSCs) [14]. The study of LSCs is the key to solving the problem of corneal epithelial regeneration. In this study, we focused on the transcription and differentiation signals of aLSCs and qLSCs. The differentiation trajectory from qLSCs to aLSCs to superficial cells was consistent with the dynamics of corneal limbal epithelial cells. In addition, we found that compared to the RNA rate of aLSCs, the response of qLSCs at 24 h after wound was relatively inert, which may be related to the position of the two distinct compartments of stem cells at the corneal limbus. aLSCs was located on the inner side of the corneal limbus and can quickly differentiate into superficial cells to supplement the loss of corneal epithelium in the face of wound, while qLSC was located on the outer side of the corneal limbus and gradually extends into the cornea during wound healing, which was like the study by [61].

Our discovery of the key transcription factor, *Creb5,* was a complete surprise. Through transcriptional signal analysis of aLSCs, qLSCs, MtCs and CBCs, it was found that the expression of *Creb5* increased during wound healing. Previous studies showed that *Creb5* promotes joint formation and the subsequent development of articular chondrocytes by driving the expression of signaling molecules [66, 101]. Furthermore, the cooperative regulation of TGF-β signaling and *Creb5* controls pharyngeal muscle development [102]. Hence one can see that *Creb5* can regulate cell proliferation and migration. Do the dynamic changes in *Creb5* expression during corneal epithelial injury repair mean that *Creb5* affects the mobilization of stem cells to participate in injury repair? So, we knocked down the expression of *Creb5* by subconjunctival injection of AAV-*Creb5*-RNAi and found that it delayed epithelial healing speed and reduced stemness and proliferation of LSCs. These results confirmed our hypothesis about the role of *Creb5*. However, we still have some limitations and shortcomings, and further research is needed to elucidate the underlying mechanisms involved in *Creb5*.

The regulation of LSC proliferation, migration, and differentiation intricately depends on the orchestration within the limbal niche microenvironment. This localized microenvironment, known as the stem cell niche, plays a pivotal role in promoting and safeguarding the stem cell populations [103–105]. Within the LSC niche, a sheltered milieu is provided to shield LSCs from excessive stimuli [106–109]. Should any pathological disruptions impact the LSC niche, it can lead to dysfunction in LSCs. In recent times, substantial progress has been achieved in delving into the limbal niche’s role in regulating LSCs. Multiple significant interactions have been uncovered between LSCs and factors governing immune cell activity [18, 110]. T cells, operating within the limbus niche, fulfill functions in maintaining quiescence, regulating epithelial thickness, and participating in wound healing processes involving corneal stem cells [14]. Melanocytes present in the limbal niche contribute to safeguarding LSPCs against UV-induced oxidative damage by facilitating the transfer of melanosomes and mitigating oxidative stress [15, 111]. However, a majority of existing investigations have predominantly centered on homeostatic conditions. Consequently, we compared the communication changes between immune cells and LSCs (aLSCs/qLSCs) under wound and homeostasis and identified that some receptor-ligand pairs showed signal enhancement after wound, such as *SPP1-CD44, Jag1-Hes1 and Birc5-Cebpb*, which were all associated with cell proliferation and tissue regeneration, indicating that immune cells regulate the process of LSCs repair of corneal epithelium. While we have identified receptor-ligand pairs that exhibit heightened activity post-wound, further experimentation is required to unveil the precise stepwise mechanism underlying these interactions.

Gaining a holistic comprehension of the intricate orchestration and their niche of LSCs during corneal healing is imperative for the continual advancement of more efficacious treatments targeting corneal blindness. In essence, our study furnishes an all-encompassing single-cell transcriptional atlas of mouse corneal homeostasis and wound healing, used to interpret the alterations of aLSCs and qLSCs behavior and their niche regulation during wound healing, providing new insights for the involvement of LSCs in corneal epithelial wound healing, and identifying new biological targets for the treatment of clinical diseases related to corneal epithelial wound.

## Conclusions

In conclusion, we performed single-cell RNA sequencing on corneal tissues from normal mice and corneal epithelium defect models, and identified the dynamics of LSC and niche cell populations during corneal epithelial wound healing. Our comparative study identified a core transcription factor Creb5, expressed in LSCs, that was significantly upregulated after corneal epithelial injury, the loss-of-function experiments revealed that silencing *Creb5* delayed the corneal epithelial healing and LSC mobilization. Furthermore, cell–cell communication analysis revealed the vital role of immune cells in niche regulation during wound healing, and highlighted *Arg1*^+^ macrophages-derived OSM can promote corneal epithelial repair effectively. This research provides a valuable single-cell resource and reference for the discovery of mechanisms and potential clinical interventions aimed at ocular surface reconstruction, potentially explaining the cellular plasticity and niche regulations in many other epithelial tissues during regeneration process.

### Supplementary Information

**Additional file 1**: List of Genes_Calculation of signature scores.

**Additional file 2**: Supplementary Figures.

**Additional file 3**: DEGs for different cell clusters of corneas.

**Additional file 4**: DEGs for subclusters of CEPC during wound.

**Additional file 5**: Ligand-receptor pairs in Immune cells-LSCs.

## Data Availability

The scRNA-seq data generated during this study are available at GEO database with the accession ID GSE247392.

## Abbreviations

LSCs: Limbal stem cells
scRNA-seq: Single-cell RNA sequencing
aLSCs: Active limbal stem cells
qLSCs: Quiescent limbal stem cells
TF: Transcription factor
UW: Un-wounded
W: Wounded
CEpCs: Corneal epithelial cells
CSCs: Corneal stromal cells
CEnCs: Corneal endothelial cells
Mono: Monocyte lineage
T: T cell
Neu: Neutrophil
GO: Gene ontology
DEGs: Differentially expressed genes
UMAP: Uniform manifold approximation and projection
CBCs: Corneal basal cells
MtCs: Mitotic cells
CSbCs: Corneal suprabasal cells
CSfCs: Corneal superficial cells
LSfCs: Limbal superficial cells
CjBCs: Conjunctival basal cells
CjSfCs: Conjunctival superficial cells
SCENIC: Single-cell regulatory network inference and clustering
IRFs: Interferon regulatory factors
LSPCs: Limbal stem/progenitor cells
AAV: Adeno-associated virus
t-SNE: T-distributed stochastic neighbor embedding
Mac: Macrophage
Lans: Langerhans cells
DCs: Dendritic cells
Tregs: Regulatory T cells
γδTs: γδT cells
OSM: Oncostatin M
