# Supplementary material for: Decoding cellular plasticity and niche regulation of limbal stem cells during corneal wound healing
Source: Stem Cell Res Ther. 2024 Jul 6;15:201. doi: 10.1186/s13287-024-03816-y (PMC11227725; doi:10.1186/s13287-024-03816-y)
Supplement: Supplementary file 2 — Additional file 2: Supplementary Figures. [file 13287_2024_3816_MOESM2_ESM.docx]

**Supplementary figures and legends**


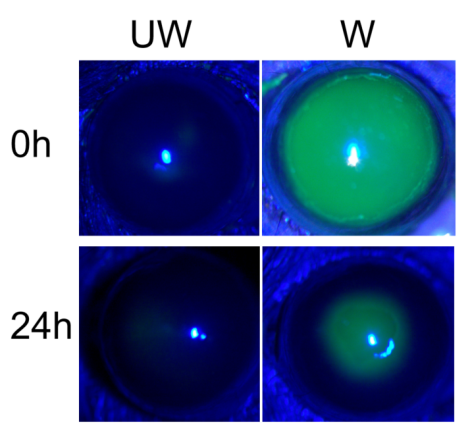


**Fig S1** Representative images showing the corneal phenotypes in mice with wounded (W) and unwounded (UW) corneal epithelium. Staining with fluorescein dye following 24-hour wound healing.


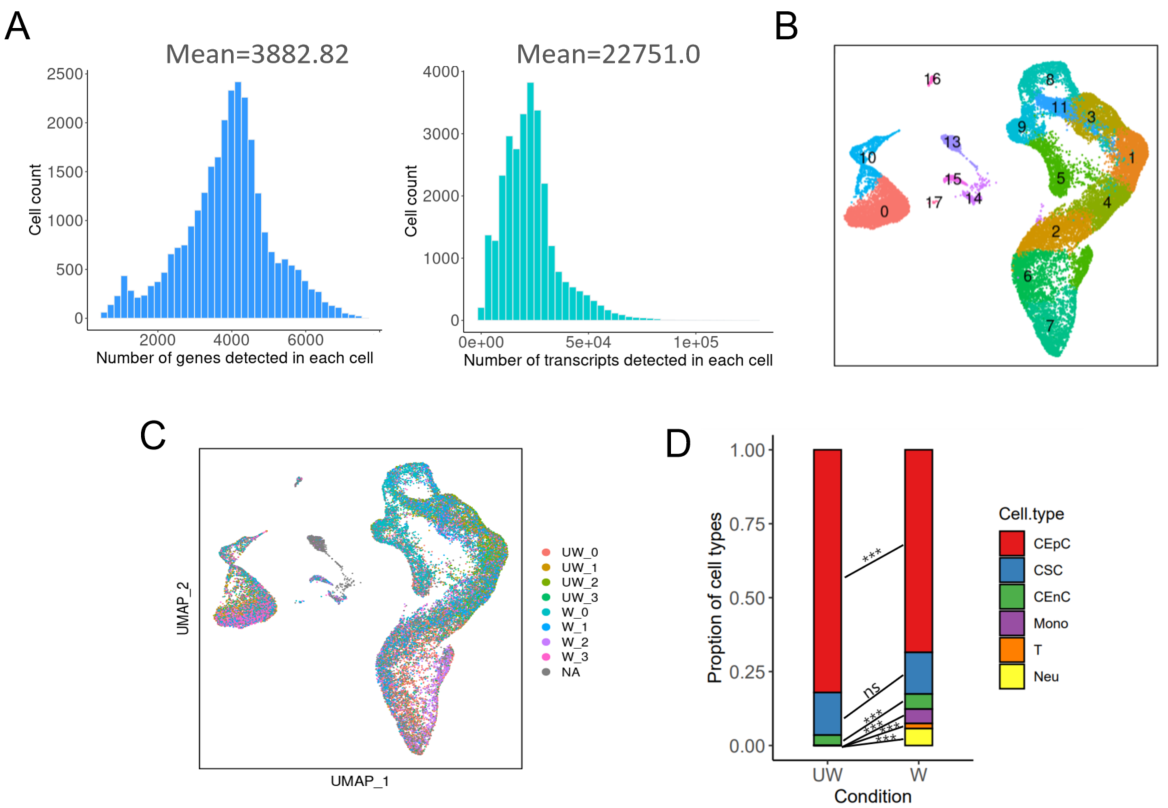


**Fig S2** Quality control and sample information of single-cell data used in this study. **A** Histograms displaying the number of genes(left) and transcripts(right) detected in individual cell. **B** UMAP plot showing 18 clusters distribution. **C** UMAP plot showing the cell distribution from 8 samples. **D** Barplot showing the proportional changes in cell types between UW and W groups. The difference between two groups was determined by chi-square test. *** p < 0.001.


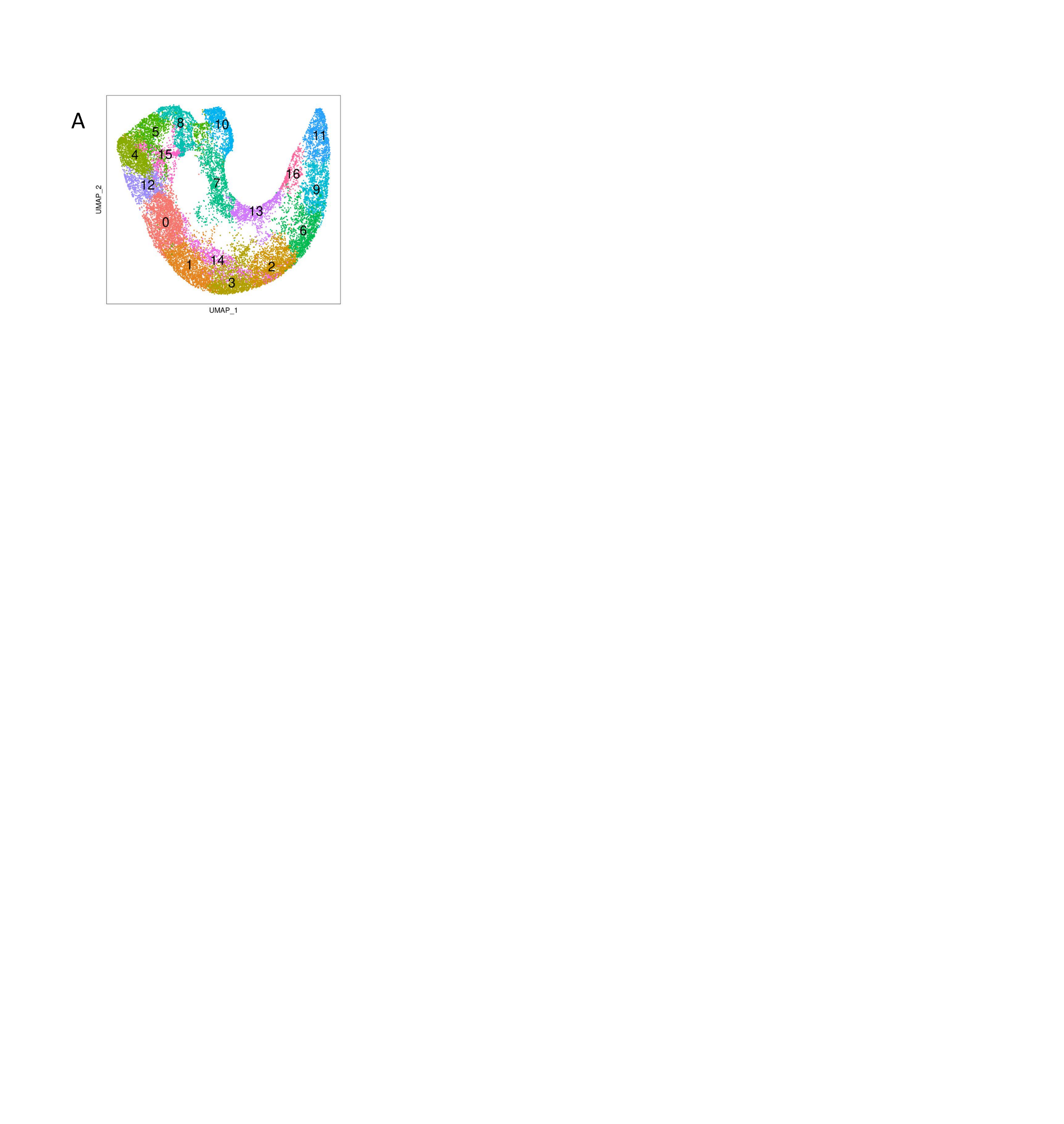


**Fig S3** UMAP plot showing 17 clusters distribution of corneal epithelial cells.


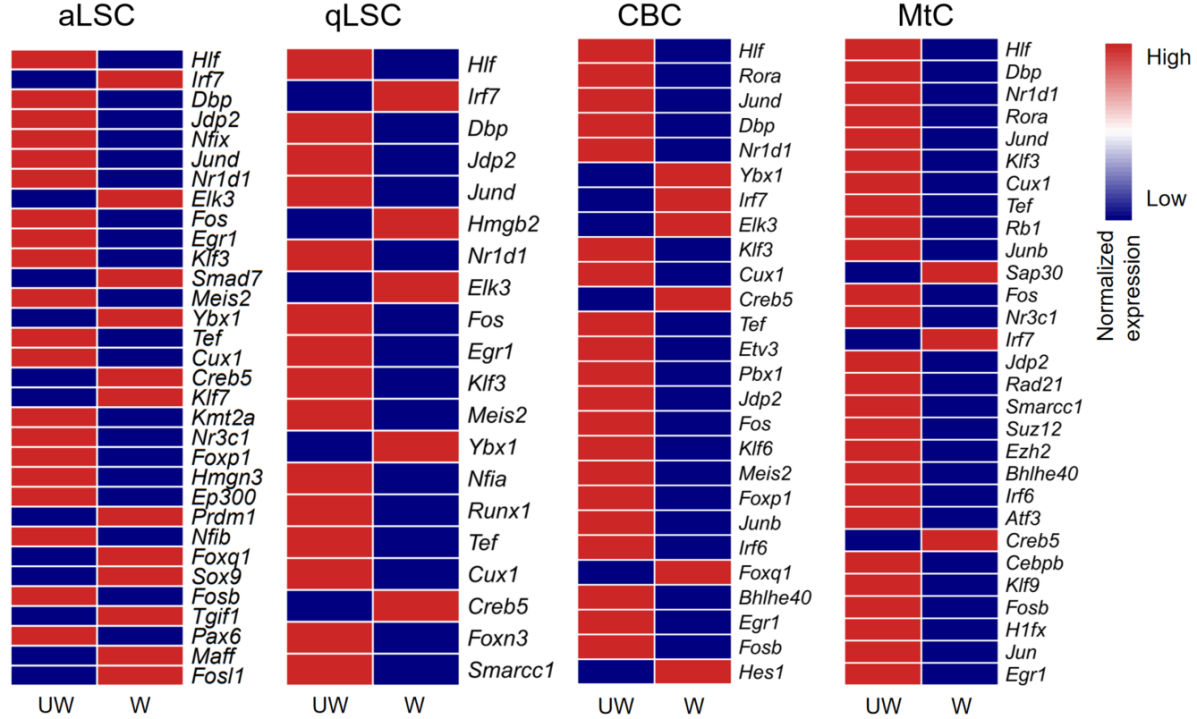


**Fig S4** Heatmaps showing the expression of inferred TFs between UW and W groups in various cell types as indicated.


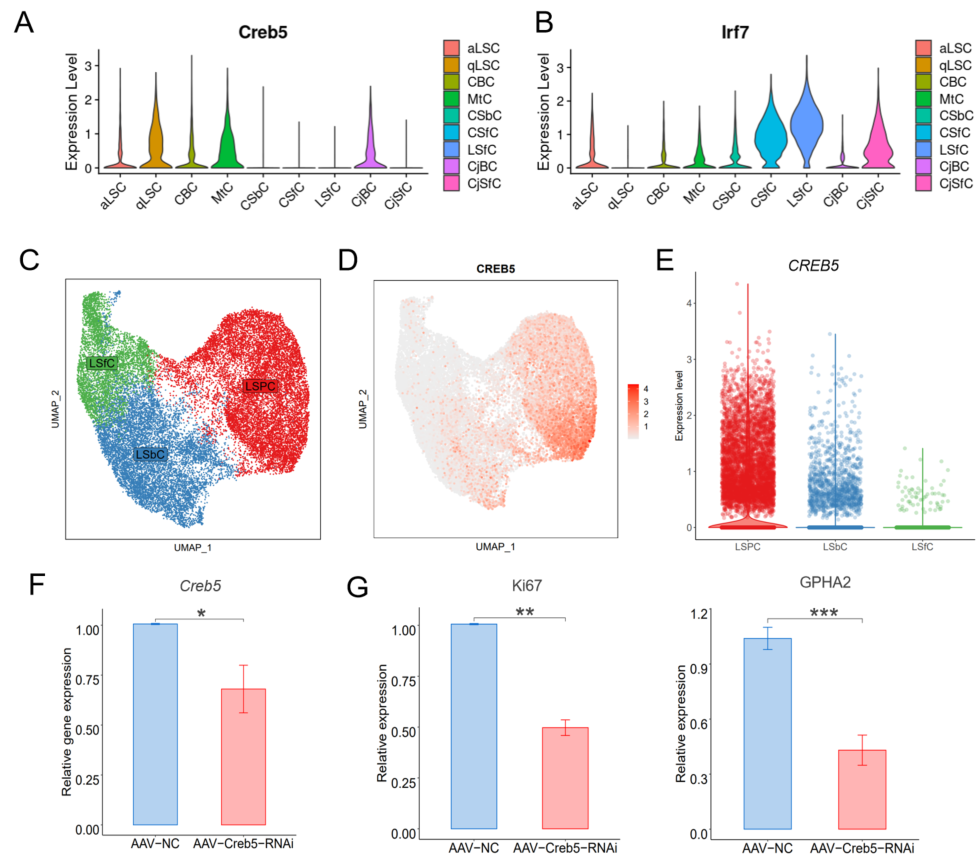


**Fig S5** Expression of *Creb5* in corneal epithelial subtypes of mouse and human. **A** Violin plot showing the expression levels of *Creb5* in CEpC subpopulations. **B** Violin plot showing the expression levels of *Irf7* in CEpC subpopulations. **C** UMAP plot showing 3 celltypes in human corneal epithelium. LSPC, limbal stem/progenitor cell; LSbC, limbal suprabasal cell; LSfC, limbal superficial cell. **D** Feature plot showing the expression of *CREB5* in human corneal epithelium. **E** Violin plot showing the expression levels of *CREB5* in human CEpC subpopulations. **F** Barplot showing the expression of *Creb5* in AAV-NC and AAV-*Creb5*-RNAi mice corneal epithelium quantified by RT-qPCR. *P < 0.05, t test. **G** Barplot showing the expression of Ki67 (left) and GPHA2 (right) in AAV-NC and AAV-*Creb5*-RNAi mice corneal epithelium. **P < 0.01, t test. *** p < 0.001.


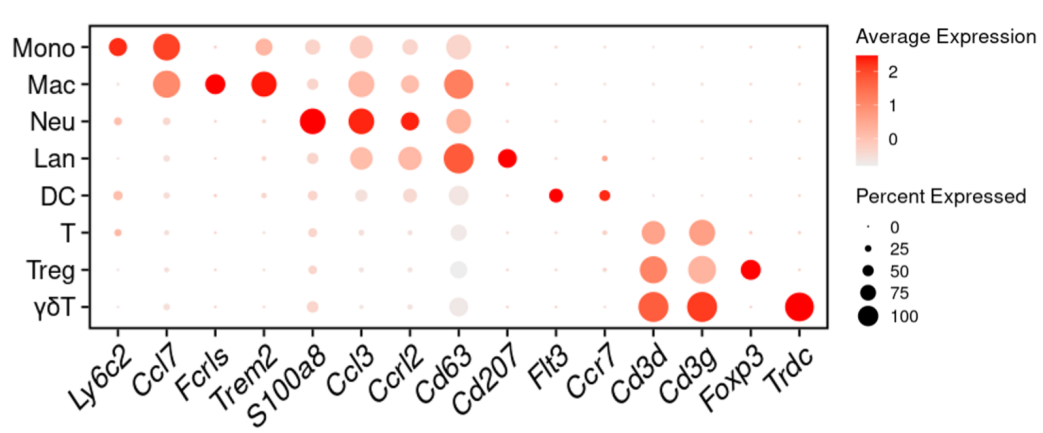


**Fig S6** Dot plot showing expression of classical marker genes for immune cell types.


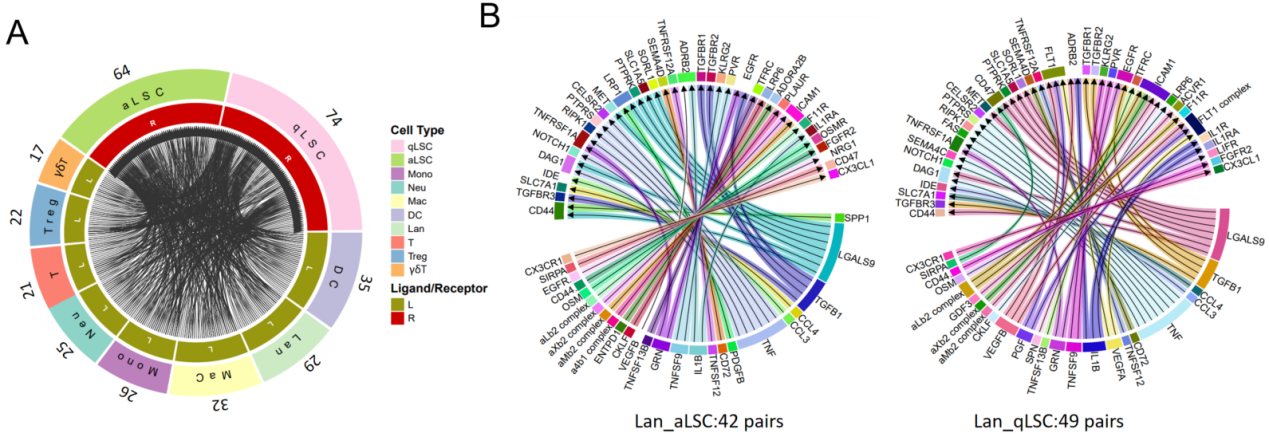


**Fig S7** Cell-cell communications between immune cells and LSCs. **A** Visualized circos showing the cell-cell communications pairs between immune cells as ligand and LSCs as receptor. The compartment represents the cell type, and its size is positively correlated with the number of ligand-receptor pairs. The number above the compartment is the number of ligand-receptor pair. **B** Chord plots showing cellular interactions between Lans and aLSCs (left)/qLSCs (right), separately. The cell types and interaction pairs number are labeled.


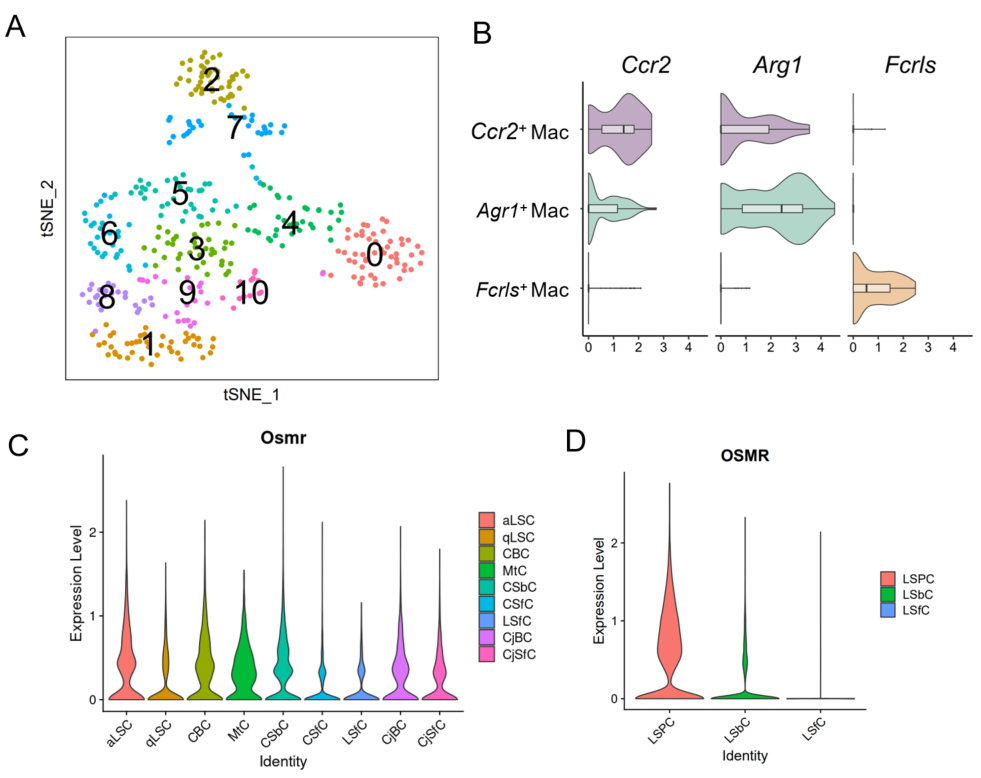


**Fig S8** Subtypes of Macs were identified and the expression of *Osmr* in cornea*.* **A** t-SNE plot showing 11 clusters distribution of Monos and Macs. **B** Violin plot showing the marker gene expression levels of three Macs subpopulations. **C** Violin plot showing the expression levels of *Osmr* in mouse CEpC subpopulations. **D** Violin plot showing the expression levels of *OSMR* in human CEpC subpopulations.
